# Clinical impact of creatine phosphokinase and c-reactive protein as predictors of postgastrectomy complications in patients with gastric cancer

**DOI:** 10.1186/s12885-021-07801-z

**Published:** 2021-01-23

**Authors:** Keishi Okubo, Takaaki Arigami, Daisuke Matsushita, Takashi Kijima, Masataka Shimonosono, Yoshikazu Uenosono, Shigehiro Yanagita, Hiroshi Kurahara, Shinichiro Mori, Takao Ohtsuka, Shoji Natsugoe

**Affiliations:** 1 Department of Digestive Surgery, Breast and Thyroid Surgery, Kagoshima University Graduate School of Medical and Dental Sciences, 8-35-1 Sakuragaoka, Kagoshima, 890-8520 Japan; 2 Department of Onco-biological Surgery, Kagoshima University Graduate School of Medical and Dental Sciences, Kagoshima, Japan

**Keywords:** CPK ratio, CRP, Gastric cancer, Complication

## Abstract

**Background:**

Postoperative complications have been linked to the morbidity and mortality of several cancers. However, predicting whether complications will occur in the early period after surgery or not is challenging. Hence, this study aimed to examine the diagnostic accuracy of serum creatine phosphokinase (CPK) and c-reactive protein (CRP) in predicting the development of postgastrectomy complications.

**Methods:**

We retrospectively analyzed 188 patients with gastric cancer (GC) who underwent gastrectomy. The diagnostic accuracy of serum CPK and CRP was investigated using the areas under the curves (AUC). The CPK ratio was defined as the CPK on postoperative day (POD) 1 to the CPK on a preoperative day.

**Results:**

Out of 188 patients, 48 (25.5%) developed postoperative complications. The complications group had a greater operative time (*p* = 0.037), higher CPK ratio on POD1 (*p* < 0.0001), and a higher serum CRP level on POD3 (*p* = 0.001). The AUC for the CPK ratio was 0.772, with an optimal cutoff value of 7.05, whereas that for CRP was 0.659, with an optimal cutoff value of 11.4 mg/L. The CPK ratio on POD1 (*p* < 0.0001) and the CRP on POD3 (*p* = 0.007) were independent factors for predicting the development of postgastrectomy complications. The CPK ratio on POD1 and the CRP on POD3 predicted postgastrectomy complications in 41 patients (85.4%). According to combined value of both CPK ratio and CRP level, the positive predictive value and the negative predictive value was 0.70 and 0.829. And sensitivity and specificity were 0.438 and 0.936.

**Conclusion:**

The CPK ratio on POD1 and the CRP on POD3 after gastrectomy for GC were predictive factors for complication development and may be employed to prevent the development of such complications and improve the prognosis of patients with GC.

**Supplementary Information:**

The online version contains supplementary material available at 10.1186/s12885-021-07801-z.

## Background

Gastric cancer (GC) is the fourth most common malignancy worldwide and the most common in East Asia [1]. Since gastrectomy with D2 lymph node dissection became the standard procedure for GC worldwide, patient outcomes have improved. Although gastrectomy is curative, postoperative complications remain a clinical issue. In a nationwide survey in Japan, the overall morbidity and mortality rates after GC resection were recently 14.2 and 1.1% for distal gastrectomy and 21.5 and 2.3% for total gastrectomy, respectively [2].

A correlation was reported between postoperative complications and negative prognosis [3–5], as well as between anastomotic leakage and recurrence rates [6, 7], in patients with GC. However, postoperative complications are often diagnosed only after the patient develops clinical symptoms. The prognosis and recurrence rates may be improved if patients who are at risk are identified in earlier stages. As supported by an increasing number of studies, high serum levels c-reactive protein (CRP) levels after gastrointestinal cancer surgery predict the clinical diagnosis of postoperative infectious complications [8–11]. Serum CRP levels on postoperative day (POD) 3 or 4 can be potentially used as an early predictor of postoperative complications [12–14]. In animal models, creatine phosphokinase (CPK) serves a biomarker of ischemic disease [15]. CPK reflects not only ischemic changes but also inflammation in muscle layers. High CPK levels have been recently identified as a risk factor for major complications related to the use of a gastric conduit for esophageal resections [16]. In this retrospective study, we aimed to examine the potential of CPK and CRP as early predictive markers of postoperative complication development in GC.

## Methods

### Patients

We retrospectively collected the data of 368 patients with GC who underwent gastrectomy between January 2010 and November 2018 in the Department of Digestive Surgery, Kagoshima University. In this study, patients with distal pancreatectomy were excluded and with cholecystectomy and splenectomy were included. Because pancreatectomy is invasive and high risk of complication surgery. After excluding those patients without CPK or CRP values and with transverse abdominal incision because the surgical trauma of transecting the rectus muscles would be expected to increase the release of CPK, we ultimately analyzed 188 patients (Table 1). Because we did not measure serum CPK and CRP levels on POD3 from period in 2011 and 2012, some patients did not have these levels drawn. Patients were grouped and staged according to the TNM classification of gastric carcinoma established by the International Union for Cancer Control [17]. The postoperative complications were evaluated according to the Clavien–Dindo Classification of Gastric Carcinoma [18]. When two or more complications occurred in one patient, the one with a higher or the highest grade was used. The serum levels of CRP and CPK were measured within 1 month preoperatively and on POD 1 and 3. In this study, the CPK ratio was defined as the CPK on POD1 to the CPK on a preoperative day. We analyzed the CRP on POD3 not on POD1 because the previous report showed that CRP measurements on POD3 and POD4 predict surgical-site infectious complications. Dutta S et al. reported CRP levels on POD3 and 4 were clinically more useful in predicting surgical site infectious complications than POD1 [9]. Furthermore, we analyzed CPK on POD1 not on POD3 because the CPK data were not obtained in 20 patients. And thus we could not obtained adequate numbers of patients with CPK levels on POD3 to obtain a statistically relevant analysis.

**Table 1:** Patient characteristics

Characteristics	Values
Age (mean)	68.0 (33–92)
Sex (male/female)	134/54
BMI	23.11 ± 12.34
TNM Stage (I/II/III/IV)	115/42/28/3
Blood loss, ml (mean)	394.2
Surgery time, min (mean)	409.3
Open surgery/Laparoscopic surgery	72/116
Surgical procedures	
TG/LATG	40/7
DG/LADG	24/76
PG/LAPG	8/33

*BMI* body mass index, *TG* total gastrectomy, *DG* distal gastrectomy, *PG* proximal gastrectomy

### Assessment of complications

We collected patient characteristics from a prospective surgical database. We have the database and established system controlled and entered by medical doctors. The database has been regularly renewed every one month. Postoperative complications such as anastomotic leakage, chylous leakage, abdominal abscess/fluid collection, pancreatic fistula, wound infection, intestinal obstruction, lung complications, and intra-abdominal bleeding were evaluated. Complications graded as II or higher were identified as major complications.

Anastomotic leakage was assessed by fluid status from drain, computed tomography (CT), or fluoroscopy in the present study. Chylous leakage indicates output of milky-colored fluid from a drain or drain site.

### Statistical analysis

To establish an appropriate CPK ratio and CRP, we conducted receiver operating characteristic (ROC) analysis. Diagnostic accuracy was assessed by determining the area under the ROC curve (AUC). We also determined the optimal cutoff values by using the AUC with Youden’s index. Using the chi-square test, we analyzed the relationships between the CPK ratio, CRP, and clinicopathological characteristics. Independent risk factors associated with the prediction of major complications were identified by logistic regression analysis. Accordingly, odds ratios and 95% confidence intervals (CI) were estimated. In addition, clinicopathological variables were analyzed using Pearson’s chi-square test. All statistical data were calculated using the SAS/JMP statistical software (version 14: SAS Institute. Inc., Cary, NC). A *p* value of < 0.05 indicated significance. Using combined values of CRP and CPK, we calculated the positive predictive value and negative predictive value.

## Results

### Patient clinicopathological characteristics

**Fig. 1:**
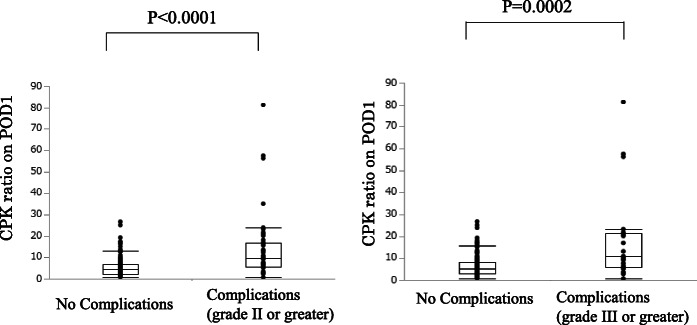
Differences in the creatine phosphokinase (CPK) ratio on postoperative day 1 (POD1) between the complications (grade II or greater and grade III or greater) and no-complications groups after gastrectomy

**Fig. 2:**
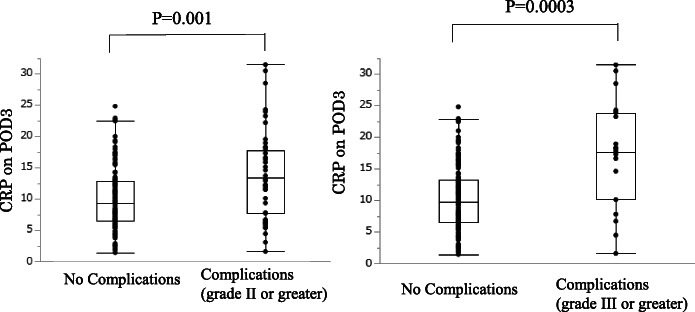
Differences in c-reactive protein (CRP) on postoperative day 3 (POD3) between the complications (grade II or greater and grade III or greater) and no-complications groups after gastrectomy

Table 1 summarizes the clinical characteristics of the 188 study patients, and Table 2 lists the levels of CPK ratio and CRP in patients with complications. Out of 188 patients, 48 (25.5%) had postoperative complications of grade II or higher (anastomotic leakage = 10, lung complications = 7, chylous leakage = 6, abdominal abscess/fluid collectio*n* = 6, pancreatic fistula = 5, wound infectio*n* = 5, intestinal obstruction = 5, intra-abdominal bleeding = 2, and anastomotic stricture = 2). The average of CPK at the preoperative time and POD1 was 82.6 U/L (complication group: 78.1 U/L vs. no-complication group: 86.5 U/L) and 527.7 U/L (complication group: 772.6 U/L, no-complication group: 443.7 U/L), respectively. These results indicated that the CPK level on POD1 was significantly higher in complications group than no-complications group (*p* = 0.001). The complications group had a higher CPK ratio on POD1 (13.83 ± 2.14) than the no-complications group (5.76 ± 0.40) (*p* < 0.0001) (Fig. 1). The complications group also had a significantly higher CRP on POD3 (13.81 ± 1.00) than the no-complications group (10.12 ± 0.43) (*p* = 0.001) (Fig. 2). Out of 188 patients, 21 (11.1%) had postoperative complications of grade III or higher (anastomotic leakage = 8, lung complications = 3, chylous leakage = 2, abdominal abscess/fluid collection = 1, pancreatic fistula = 2, intestinal obstruction = 3, intra-abdominal bleeding = 1, and anastomotic stricture = 1).

**Table 2:** The levels of CPK ratio and CRP in patients with complications

Complications (*n* = 48)	CPK ratio	CRP
anastomotic leakage (*n* = 10)	19.1	18.5
lung complications (*n* = 7)	8.21	14.2
chylous leakage (*n* = 6)	7.98	7.13
abdominal abscess/fluid collection (*n* = 6)	8.87	13.4
pancreatic fistula (*n* = 5)	23.4	15.8
wound infection (*n* = 5)	11.2	13.2
intestinal obstruction (*n* = 5)	9.57	12.1
intra-abdominal bleeding (*n* = 2)	4.02	16.7
anastomotic stricture (*n* = 2)	13.7	9.02

*CPK* creatine phosphokinase, *CRP* c-reactive protein

The complications group had a higher CPK ratio on POD1 (19.24 ± 4.76) than the no-complications group (6.47 ± 0.39) (*p* = 0.0002) (Fig. 1). The complications group also had a higher CRP on POD3 (17.09 ± 1.89) than the no-complications group (10.35 ± 0.38) (*p* = 0.0003).

The CPK ratio and CRP of each complications were described in Table 2. CPK ratio were especially higher in patients with anastomotic leakage (19.1) and pancreatic fistula (23.4). In Fig. 1, four patients with high CPK ratio had anastomotic leakage (*n* = 2) and pancreatic fistula (*n* = 2). Moreover, these patients underwent Total Gastrectomy (*n* = 2), Laparoscopy-assisted distal gastrectomy (*n* = 1), and Laparoscopy-assisted proximal gastrectomy (*n* = 1).

### Relationship between the CPK ratio on POD1 and CRP on POD3 and postoperative complications

**Fig. 3:**
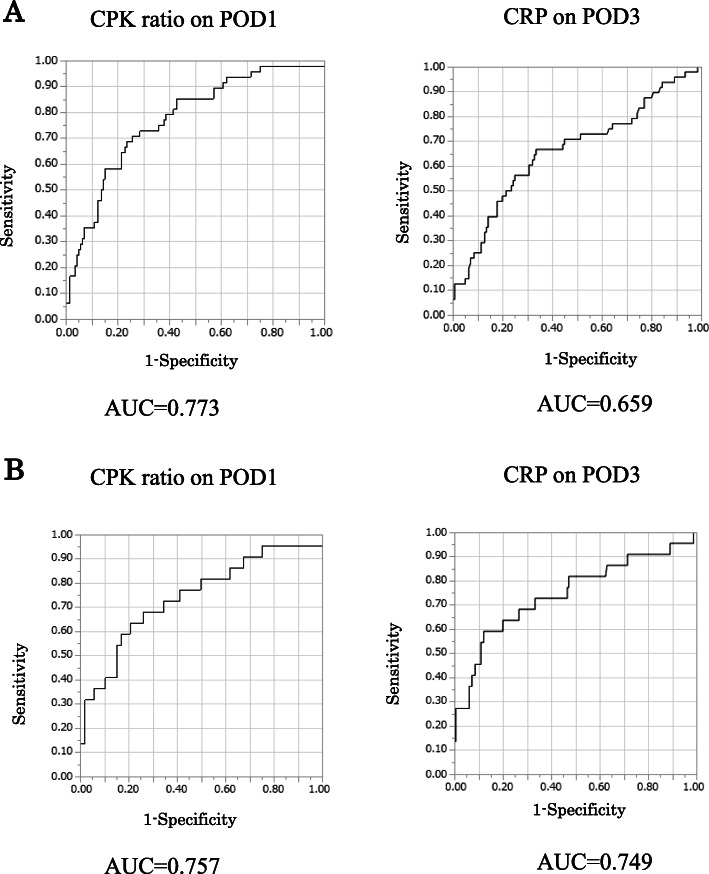
**a**. Receiver operating characteristic (ROC) curves for the diagnostic accuracy of the creatine phosphokinase (CPK) ratio on postoperative day 1 (POD1) and c-reactive protein (CRP) on POD3 to predict the development of postoperative complications (grade II or greater). **b.** Receiver operating characteristic (ROC) curves for the diagnostic accuracy of the creatine phosphokinase (CPK) ratio on postoperative day 1 (POD1) and c-reactive protein (CRP) on POD3 to predict the development of postoperative complications (grade III or greater)

We used the AUC in evaluating the diagnostic accuracy of the CPK ratio on POD1 and CRP on POD3 for predicting the development of postoperative complications of grade II or higher (Fig. 3a) and of grade III or higher (Fig. 3b). In patients of postoperative complications of grade II or higher, for the CPK ratio on POD1, the AUC was 0.773, with an optimal cutoff value of 7.05, and the sensitivity and specificity were 0.687 and 0.775, respectively (95% CI: 0.68–0.84, *p* < 0.001). According to CPK ratio on POD1, the positive predictive value and the negative predictive value was 0.50 and 0.877. For the CRP on POD3, the AUC was 0.659, with an optimal cutoff value of 11.4 mg/L, and the sensitivity and specificity were 0.667 and 0.665, respectively (95% CI: 0.56–0.75, *p* < 0.001). According to CRP on POD3, the positive predictive value and the negative predictive value was 0.367 and 0.826. In patients of postoperative complications of grade III or higher for the CPK ratio on POD1, the AUC was 0.757, with an optimal cutoff value of 9.20, and the sensitivity and specificity were 0.65 and 0.79, respectively (95% CI: 0.61–0.86, *p* < 0.001). According to CPK ratio on POD1, the positive predictive value and the negative predictive value was 0.227 and 0.950. For the CRP on POD3, the AUC was 0.749, with an optimal cutoff value of 16.6 mg/L, and the sensitivity and specificity were 0.65 and 0.881, respectively (95% CI: 0.58–0.86, *p* < 0.001). According to CRP on POD3, the positive predictive value and the negative predictive value was 0.151 and 0.917.

**Fig. 4:**
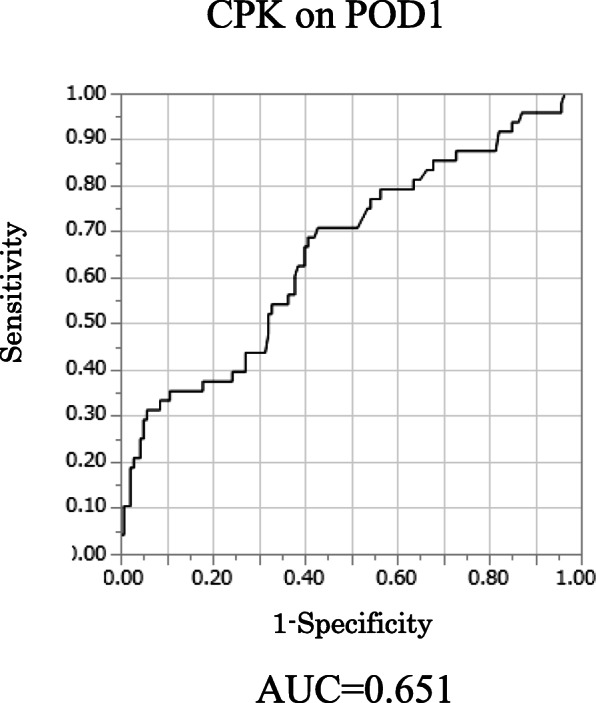
Receiver operating characteristic (ROC) curves for the diagnostic accuracy of the creatine phosphokinase (CPK) ratio on postoperative day 1 (POD1) to predict the development of postoperative complications

**Fig. 5:**
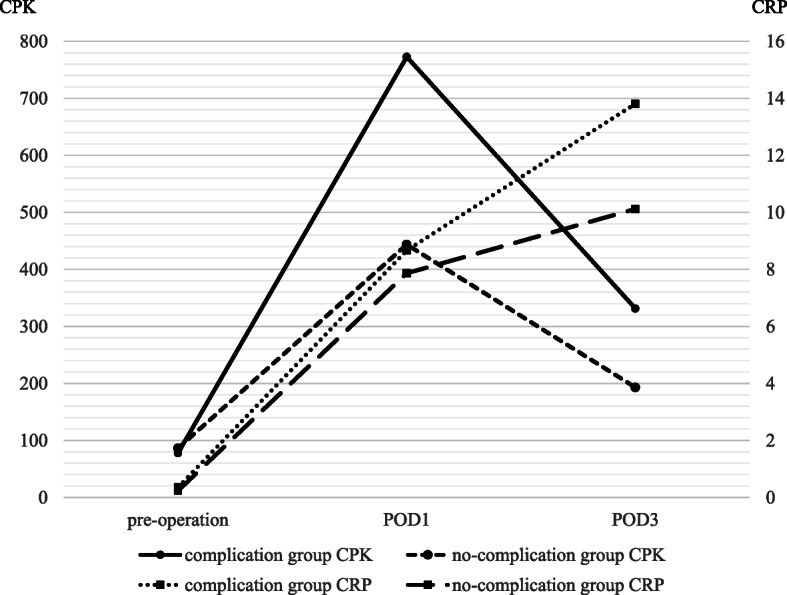
Changing pattern of CPK and CRP level

For the CPK on POD1, the AUC was 0.651, with an optimal cutoff value of 389, and the sensitivity and specificity were 0.687 and 0.593, respectively (95% CI: 0.56–0.74, *p* < 0.001) (Fig. 4). And CPK on POD1 and CRP on POD3 were higher than CPK on POD3 and CRP on POD1 (Fig. 5). Therefore, we selected the CPK ratio on POD1 and CRP on POD3 for statistical analyses.

### Univariate and multivariate analyses of clinical factors for postoperative complications

In the univariate analysis, the complications group had a significantly longer surgery time (*p* = 0.037), higher CPK ratio on POD1 (*p* < 0.0001), and higher CRP on POD3 (*p* = 0.0028) than the no-complications group. According to the multivariate analysis, the CPK ratio on POD1 (*p* < 0.0001) and CRP on POD3 (*p* = 0.007) were significant independent factors for predicting postgastrectomy complications (Table 3).

**Table 3:** Univariate analyses of clinical factors for postoperative complications

	Univariate analysis		*p*	Multivariate analysis		*p*
	No complications (*n* = 140)	Complications (*n* = 48)		Odds ratio	95% CI	
Sex						
Male	103	31	0.837			
Female	37	17				
Age						
≤ 70	74	26	0.563			
> 70	66	19				
BMI						
≤ 23	77	22	0.397			
> 23	63	24				
Surgery time (min)						
≤ 400	71	16	0.037	1.00	reference	
> 400	69	32		1.90	0.877–4.129	*p* = 0.103
Blood loss (ml)						
≤ 400	96	31	0.610			
> 400	44	17				
Preoperative chemotherapy						
Absent	111	32	0.077			
Present	29	16				
Stage						
I/II	118	39	0.628			
III/IV	22	9				
Surgical approach						
open	52	20	0.578			
laparoscopic	88	28				
Type of operation						
TG	33	14	0.444			
others	107	34				
CPK ratio on POD1						
≤ 7.05	107	15	< 0.0001	1.00	Reference	
> 7.05	33	33		7.67	3.585–16.44	*p* < 0.0001
CRP on POD3						
≤ 11.4	90	19	0.0028	1.00	Reference	
> 11.4	50	29		2.76	1.291–5.923	*p* = 0.007

*TG* total gastrectomy

Of the 48 patients who developed complications, 33 (68.7%) had positive CPK ratio on POD1, while 29 (60.4%) had positive CRP on POD3. Conversely, the CPK ratio on POD1 and the CPK on POD3 were negative in only 7 (14.6%) patients. Thus, the CPK ratio on POD1 and CRP on POD3 could potentially predict the development of complications in 41 (85.4%) patients after gastrectomy (Table 4). According to combined value of both CPK ratio and CRP level, the positive predictive value and the negative predictive value was 0.70 and 0.829 (Table 5). And sensitivity and specificity were 0.438 and 0.936.

**Table 4:** Relationship between the CPK ratio and CRP in patients with complications

*n* = 48	CPK ratio positive (*n* = 33)	CPK ratio negative (*n* = 15)
CRP positive (*n* = 29)	21 (43.8%)	8 (16.7%)
CRP negative (*n* = 19)	12 (25.0%)	7 (14.6%)

*CPK* creatine phosphokinase, *CRP* c-reactive protein

**Table 5:** Combined value of both CPK ratio and CRP level

*n* = 188	Complications (*n* = 48)	No- Complications (*n* = 140)
CPK ratio positive and CRP positive (*n* = 30)	21 (11.2%)	9 (4.8%)
CPK ratio negative or CRP negative (*n* = 158)	27 (14.3%)	131 (69.7%)

*CPK* creatine phosphokinase, *CRP* c-reactive protein

## Discussion

Postoperative complications may have a significantly negative impact on the recurrence and survival of patients with colorectal and esophageal cancer [19–25]. Postoperative complications, particularly anastomotic leakage, are related to negative prognosis in patients with GC [26, 27]. In the present study, we examined the potential of the CPK ratio and serum CRP to predict the development of postgastrectomy complications and found that the CPK ratio on POD1 and serum CRP on POD3 were significant independent predictive factors. The CPK ratio on POD1 and the CRP on POD3 were positive in 68.7 and 60.4% of patients who developed postgastrectomy complications, respectively. Furthermore, the combination of these two factors predicted the development of complications in 41 patients (85.4%) after gastrectomy. Thus, these factors can potentially identify patients who will develop complications, such as anastomotic leakage, abdominal abscess/fluid collection, pancreatic fistula, wound infection, and lung complications, as early as POD3. Several interventions, such as food intake cessation, antibiotic therapy continuation, and CT examinations, may be initiated in the early period to diagnose, treat, or even potentially prevent complications. Positive predictive value based on the combined analysis of both CPK ratio and CRP level was 0.70. Accordingly, we should pay attention to anastomotic leakage or pancreatic fistula in patients with both high levels. Actually we conduct gastrograffin swallow study and put off starting food, operatively placed drains removal for these patients.

We examined the CPK ratio and CRP in 188 patients after gastrectomy. CRP is a type of acute-phase protein that is produced in the liver and functions as an inflammatory mediator. Plasma CRP concentrations increase in the liver after surgical trauma and infection. Shishido et al. reported that elevated CRP levels on POD3 help physicians predict postoperative infectious complications of grade III or higher based on the Clavien–Dindo classification [13]. Lee et al. demonstrated that reductions in CRP concentrations between POD3 and POD5 and between POD2 and POD3 are the most accurate factors for predicting postoperative complication development [28]. In that study, the CRP levels were the highest on POD3. Based on these findings, we employed CRP on POD3 as a marker for predicting the development of postoperative complications. It may also be used to assess the need for additional treatments in the postoperative period.

Kobayashi et al. identified high levels of CPK and CRP on POD1 after thoracoscopic esophagectomy as risk factors for major complications related to gastric conduit [16]. However, reports supporting the efficacy of CPK for predicting the development of postoperative complications are limited. The present study is the first to show that CPK and CRP are both useful for predicting the development of postgastrectomy complications. According to the combined analysis of both CPK ratio and CRP level, the positive predictive value and the negative predictive value were high in Table 5. Hence, we believe that the combined analysis of both CPK ratio and CRP level has the clinical utility for predicting postoperative complications. In general, the serum CPK level elevates rapidly in patients with acute myocardial infarction, autoimmune myositis, muscular dystrophy, and acute renal injury [29–31]. Given that preoperative CPK levels varied among our patients, the CPK ratio was defined in our study as the CPK on POD1 to preoperative CPK. According to the AUC, the CPK ratio was more accurate than POD1 CPK (Supplementary Figure 1). Moreover, the CPK ratio was higher in patients who required transverse incisions than those who required midline incision (average of CPK ratio; 29.1 and 7.71); hence, we excluded those who required transverse incision from the study population. Transecting the rectus muscle would be expected to increase release of CPK. Meanwhile, no significant difference was observed between open and laparoscopic surgery (average of CPK ratio; 7.7 and 8.0, *p* = 0.57). Therefore, the cutoff value for the POD1 CPK ratio may be beneficial for both open surgery by midline incision and laparoscopic surgery. In this study, the CPK ratio was higher in patients with anastomotic leakage and pancreatic fistula. And patients with chylous leakage and intra-abdominal bleeding had lower CPK ratio. According to these results, CPK ratio might be affected by organ damage and ischemia. Since it is guessed patients have severe organ damage and ischemic change on POD1, the CPK level was higher in POD1 than POD3.

The present study, however, has several potential limitations. Considering that this study is retrospective in design and was conducted only in a single institution, further large prospective studies are needed to validate the results obtained and confirm whether the early prediction of complications and prevention based on an elevated CPK ratio on POD1 and CPR on POD3 improve postoperative outcomes and reduce the morbidity especially for anastomotic leak, pancreatic fistula and mortality rates after gastrectomy. Generally, morbidity rate after gastrectomy in Japan was lower, comparing with other countries. Furthermore, the morbidity rate may be affected by low body mass index, fewer co-morbidities and surgical technique. These finding suggested that CPK ratio and CRP is higher in other countries. Accordingly, further large studies including patients in other countries are needed to strengthen our conclusions. In this study, because the majority of the patients were operated within the last 3 years, any survival statistics would be premature and therefore we are not reporting the effects of these values on survival. Prospective studies should investigate whether early diagnostic or therapeutic approaches based on elevated serum CPK ratio and CRP actually lead to earlier detection of postoperative complications, improved outcomes, and reduced morbidity after gastrectomy.

In the present study, the combination of the CPK ratio on POD1 and CRP on POD3 accurately predicted the development of postoperative complications. If the CPK ratio on POD1 or CRP on POD3 is positive, the development of complications and appropriate strategies should be considered in the early period.

## Conclusions

The CPK ratio on POD1 and the CRP on POD3 after gastrectomy can be potentially used as predictive factors for the development of postoperative complications in GC.

These values alone and especially in combination can be used to predict complications and potentially ameliorate or possibly even prevent the development of complications.

## Supplementary Information

**Additional file 1.**

## Data Availability

The datasets used and /or analyzed during the current study are available from the corresponding on reasonable request.

## Abbreviations

CPK: Creatine phosphokinase
CRP: C-reactive protein
GC: Gastric cancer
AUC: Areas under the curves
POD: Postoperative day
ROC: Receiver operating
CI: Characteristic confidence intervals
BMI: Body mass index
TG: Total gastrectomy
DG: Distal gastrectomy
PG: Proximal gastrectomy
