# Supplementary figures and images for: Clinical impact of creatine phosphokinase and c-reactive protein as predictors of postgastrectomy complications in patients with gastric cancer
Source: BMC Cancer. 2021 Jan 23;21:95. doi: 10.1186/s12885-021-07801-z (PMC7825180; doi:10.1186/s12885-021-07801-z)

## Slide 1
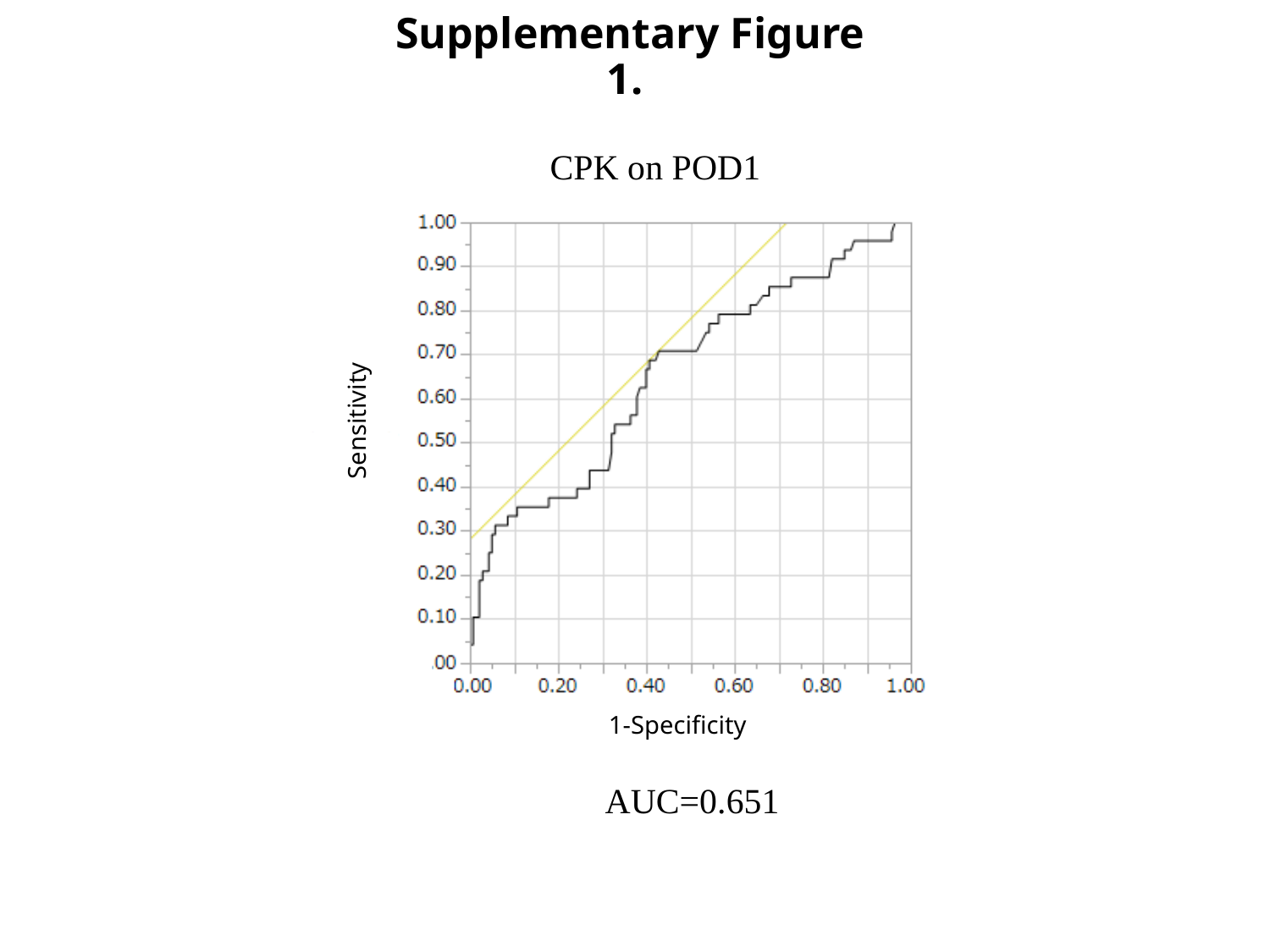

Supplementary Figure 1.
CPK on POD1
Sensitivity
1-Specificity
AUC=0.651

Supplement: Supplementary file 1 — Additional file 1. [file 12885_2021_7801_MOESM1_ESM.pptx]
